# Impact of BCR-ABL1 Transcript Type on Outcome in Chronic Myeloid Leukemia Patients Treated With Tyrosine Kinase Inhibitors: A Pairwise and Bayesian Network Meta-Analysis

**DOI:** 10.3389/fonc.2022.841546

**Published:** 2022-02-10

**Authors:** Kangkang Chen, Yingying Ruan, Kewei Tian, Peisheng Xiong, Nan Xia, Jin Li, Wen Huang, Feiyan Cao, Qifeng Chen

**Affiliations:** ^1^Department of Non-communicable Diseases Control and Prevention, Shaoxing Center for Disease Control and Prevention, Shaoxing, China; ^2^Department of General Practice, Shaoxing People’s Hospital, Shaoxing, China; ^3^School of Public Health, Hangzhou Medical College, Hangzhou, China; ^4^Immunization Program Section, Zhanggong District Center for Disease Control and Prevention, Ganzhou, China; ^5^The First Affiliated Hospital, Zhejiang University, Hangzhou, China; ^6^Emergency Department, Shaoxing Hospital of Traditional Chinese Medicine, Shaoxing, China

**Keywords:** chronic myeloid leukemia (CML), tyrosine kinase inhibitor (TKI), BCR-ABL1, e13a2, e14a2

## Abstract

**Purpose:**

To evaluate the impact of BCR-ABL1 transcript type on outcome in chronic myeloid leukemia (CML) patients treated with tyrosine kinase inhibitors (TKIs).

**Methods:**

PubMed, Embase and Cochrane library were systematically searched for relevant studies. Outcomes assessed were: major molecular response (MMR) at 6, 12, 18 and 60 months, deep molecular response (DMR) at 6, 12, 18 and 60 months, event-free survival (EFS), progression-free survival (PFS), overall survival (OS) and treatment-free remission (TFR). Odds ratios (ORs) and hazard ratios (HRs) were estimated and pooled using a random effect model.

**Results:**

A total of 16 retrospective cohort studies involving 5,411 patients were included in this study. Compared with e13a2 transcripts, there was a statistically significant advantage for patients with e14a2 (alone or with co-expressed e13a2) in terms of MMR and DMR at 6, 12 and 18 months. This benefit was sustained up to 5 years for patients with e14a2 transcripts (OR 1.60, 1.23-2.07 and 2.21, 1.71-2.87, respectively), but not for patients with both transcripts. The expression of e14a2 also improved EFS (HR 0.71, 0.53-0.94) and OS (HR 0.76, 0.57-1.00) throughout treatment period. Importantly, having e14a2 transcripts were associated with a higher rate of TFR (OR 2.94, 1.70-5.08) in CML patients attempting TKI discontinuation. Bayesian network meta-analysis showed that e14a2 had the highest probability to be the most favorable transcript type for all outcomes, followed by both and e13a2.

**Conclusions:**

The expression of e14a2 had a positive impact on MMR, DMR, EFS, OS and TFR. We suggest that in the future, the e14a2 transcript can be added to the list of prognostic factors to guide clinical decisions in treating CML.

**Systematic Review Registration:**

[https://www.crd.york.ac.uk/PROSPERO/#myprospero], identifier PROSPERO (CRD42021288440).

## Introduction

A reciprocal translocation between chromosomes 9 and 22 results in the fusion gene *BCR-ABL1*, which is the genetic hallmark of chronic myeloid leukemia (CML) (1, 2). The breakpoints of *BCR* gene cluster occur primarily within a 5.8-kb region known as the major breakpoint cluster region (*M-BCR*) that spans exons e12-16 (historically named b1-5); the breakpoints in the *ABL1* gene are similarly variable (3). Of note, e13 or e14 is more prone to fuse with ABL exon 2 (a2), giving rise to the e13a2 or the e14a2 transcripts. According to statistics, more than 90% of CML patients carry either the e14a2 or the e13a2 transcript alone. The co-expression of both transcripts (e14a2 and e13a2) can also be found in approximately 5-10% of patients. Both transcripts are translated into constitutively active proteins of 210 kDa which serve as targets for tyrosine kinase inhibitors (TKIs). The life expectancy of CML patients who may once have died within 7 years of diagnosis in the pre-TKI era is now more likely close to that of general population (4, 5). However, nearly 40% of CML patients treated with TKIs fail to achieve an optimal response throughout 5-year treatment period, or later relapse (6, 7). One possible hypothesis for the causes of resistance to TKIs could be due to the different protein tyrosine kinases (i.e., e13a2 and e14a2) that differ from one another by 75 base pairs. This structural difference may be related to the rates of transcription and translation, and the affinity of protein tyrosine kinases to TKIs, which may therefore affect the response to TKI treatment (8). If confirmed, transcript type could be used to guide clinical decisions in treating CML, especially at a time when treatment-free remission (TFR) is becoming the ultimate goal of therapy.

So far, the impact of BCR-ABL transcript type on outcome in CML patients has been investigated in few studies but was inconclusive in the TKI era. In three studies, no significant difference in major molecular response (MMR) was found between different transcripts (9–11), whereas seven studies found that superior MMR was observed in patients with e14a2 transcripts (12–18). Six studies reported that e14a2 was a better predictor of deep molecular response (DMR) (10, 12, 13, 15, 16, 18). However, Mulas et al. (19) and Marce et al. (11) showed that the transcript types did not affect DMR. In addition to molecular response, survival was also a major subject of debate. Event-free survival (EFS) was demonstrated to be the same in two studies (16, 18), but to be significantly better in e14a2 patients in four studies (13, 15, 20, 21). Progression-free survival (PFS) was demonstrated to be the same in three studies (11, 12, 19), but to be significantly better in e14a2 patients in two studies (13, 15). Overall survival (OS) was demonstrated to be the same in seven studies (12, 13, 16, 19–22), but to be significantly different in two (11, 15). To our knowledge, no meta-analysis has been conducted to summarize the conflicting evidence.

Given the inconsistency of the above findings, the aim of this meta-analysis is to evaluate whether the impact on response and survival in TKI-treated patients with CML varies by different transcript types (e13a2 vs e14a2 vs both).

## Methods

This meta-analysis was performed according to the PRISMA statement (Preferred Reporting Items for Systematic Reviews and Meta-Analyses) (23). The research protocol was registered and approved in PROSPERO (CRD42021288440).

### Data Sources

We searched the electronic databases (PubMed, Embase and Cochrane library) from the inception dates to October 19, 2021, using the MeSH (Medical Subject Headings) “Leukemia, Myelogenous, Chronic, BCR-ABL Positive” and text words “e13a2”, “e14a2”, “b2a2”, and “b3a2” to identify published studies evaluating the impact of typical BCR-ABL transcript type on outcome in chronic phase CML patients treated with TKIs. The detailed search strategies are shown in **Table S1**. Reference lists of included studies were also manually searched to identify any relevant studies that did not come up in the initial search. No limits were applied for language.

### Selection Criteria

Studies were included if they met the following criteria: (1) enrolling adults with chronic phase CML expressing typical BCR-ABL transcripts e13a2 (b2a2), e14a2 (b3a2), or co-expressed e13a2 (b2a2) with e14a2 (b3a2) at the beginning of the study who received frontline TKIs treatment; and (2) reporting any clinical efficacy outcomes (see below) during follow-up or providing corresponding Kaplan-Meier curves. Exclusion criteria were as follows: (1) studies in which BCR-ABL 1 transcript level was not assessed according to the International Scale (IS); (2) reviews, abstract, conference proceedings or case reports; and (3) duplicate studies from the same database (only the most recent study was included in the analysis).

Two researchers (J.L. and W.H.) independently screened the titles and abstracts to evaluate the potential studies. If a study was relevant, the full article was obtained for further reviewed by two independent reviewers (K.C. and Y.R.). Any disagreements were resolved in a consensus meeting with a third researcher (F.C.) as a referee.

### Data Extraction and Risk of Bias Assessments

Two researchers (K.C. and Y.R.) independently extracted all relevant data from the included studies using a predefined information extraction sheet. Information extracted included lead author, publication year, study design, transcript type, sample size, type of TKI therapy, criterion for DMR, median follow-up, risk of bias, patient characteristics (including sex ratio, age, Sokal score and median baseline laboratory values) and data on outcomes (see below). The extracted data were checked for accuracy by a third researcher (P.X). Any disagreements were resolved by consensus.

The Newcastle-Ottawa Scale (NOS) for observational studies was used to evaluate the risk of bias of included studies. Two researchers (K.C. and K.T.) individually evaluated study quality by examining nine items: 1) Representativeness of the exposed cohort, 2) Selection of the non-exposed cohort, 3) Ascertainment of exposure, 4) Demonstration that outcome of interest was not present at start of study, 5) Study controls for risk score, 6) Study controls for any additional factor, 7) Assessment of outcome, 8) Was follow-up long enough for outcomes to occur, and 9) Adequacy of follow up of cohorts. Each item is scored from 0 to 1, for a total maximum of 9 points. The overall methodological quality of each study can be divided into low risk of bias (7-9 points), medium risk of bias (4-6 points) and high risk of bias (≤ 3 points). Any disagreements were resolved in a consensus meeting with a third researcher (F.C.) as a referee.

### Definition of Outcomes

Primary outcomes were MMR and DMR at 60 months because achieving MMR at any time represents optimal response for CML patients, and sustained DMR is a prerequisite for TFR. Secondary outcomes were MMR at 6, 12 and 18 months, DMR at 6, 12 and 18 months, the rate of TFR, and long-term survival (EFS, PFS and OS). We chose the rate of TFR as the secondary outcome since the number of studies concerning the impact of different transcripts on TFR was limited at present. We chose EFS, PFS and OS as the secondary outcomes since no improvements were found in survival throughout treatment period in the majority of the studies (24). In our study, DMR is referred to as MR^4^ or MR^4.5^ to meet the need of pooling various studies with different DMR criteria. Definitions of response criteria were based on the European LeukemiaNet (ELN) 2020 recommendations (25).

### Statistical Analysis

The required data were extracted directly from each article. If evaluated outcomes were only presented as Kaplan-Meier curves, we used Engauge Digitizer 4.1 and the excel file provided by Tierney et al. (26)for calculating the corresponding log hazard ratios (HRs) and standard errors. Typical BCR-ABL transcripts were compared using pairwise comparison. The odds ratios (ORs), HRs and 95% confidence intervals (CIs) were calculated from the DerSimonian-Laird statistical model. Statistical heterogeneity across studies was evaluated using the *I^2^* statistic. *I^2^* < 25% reflected mild heterogeneity, 25-50% moderate heterogeneity, and > 50% severe heterogeneity. We chose a random-effects model to pool the data because of its conservative summary estimate. For primary outcomes with severe heterogeneity, we performed meta-regression and subgroup meta-analyses to explore sources of heterogeneity. To evaluate whether the effects of different transcripts on primary outcomes were affected by characteristics of the studies and patients, exploratory sub-analyses were also performed. Factors are reported only if they were statistically significant. Publication bias was estimated using Begg’s funnel plot (27).

We performed the traditional pair-wise meta-analysis with Stata version 16 (StataCorp, College Station, TX, USA). To fully leverage available data and increase confidence in our results, network meta-analysis (NMA) was done using JAGS software within R by use of rjags (R package Version 4.3.0) and gemtc (R package Version 0.8). This is a method which could pool evidence from direct and indirect comparisons within a Bayesian framework (28–30). Outcomes were calculated as ORs or HRs and reported with the 95% credible intervals (CrIs). For convergence, the first 5,000 iterations were discarded as burn-in, and the results were presented according to a further 20,000 iterations. The goodness of fit of the model was appraised with the deviance information criterion (DIC). When the DIC value of fixed-effect model or random-effect model was calculated, we chose the lower DIC model as the primary analytical model. To evaluate consistency between direct and indirect comparisons, node-splitting method was performed to compare the ORs/HRs from the NMA with corresponding ORs/HRs from traditional pair-wise meta-analysis. Finally, the surface under the cumulative ranking curve (SUCRA) analyses were performed to estimate the probability of each transcript to be the most favorable for each outcome. All tests were 2-tailed, and *P* < 0.05 was considered statistically significant.

## Results

### Studies Retrieved and Characteristics

**Figure 1:**
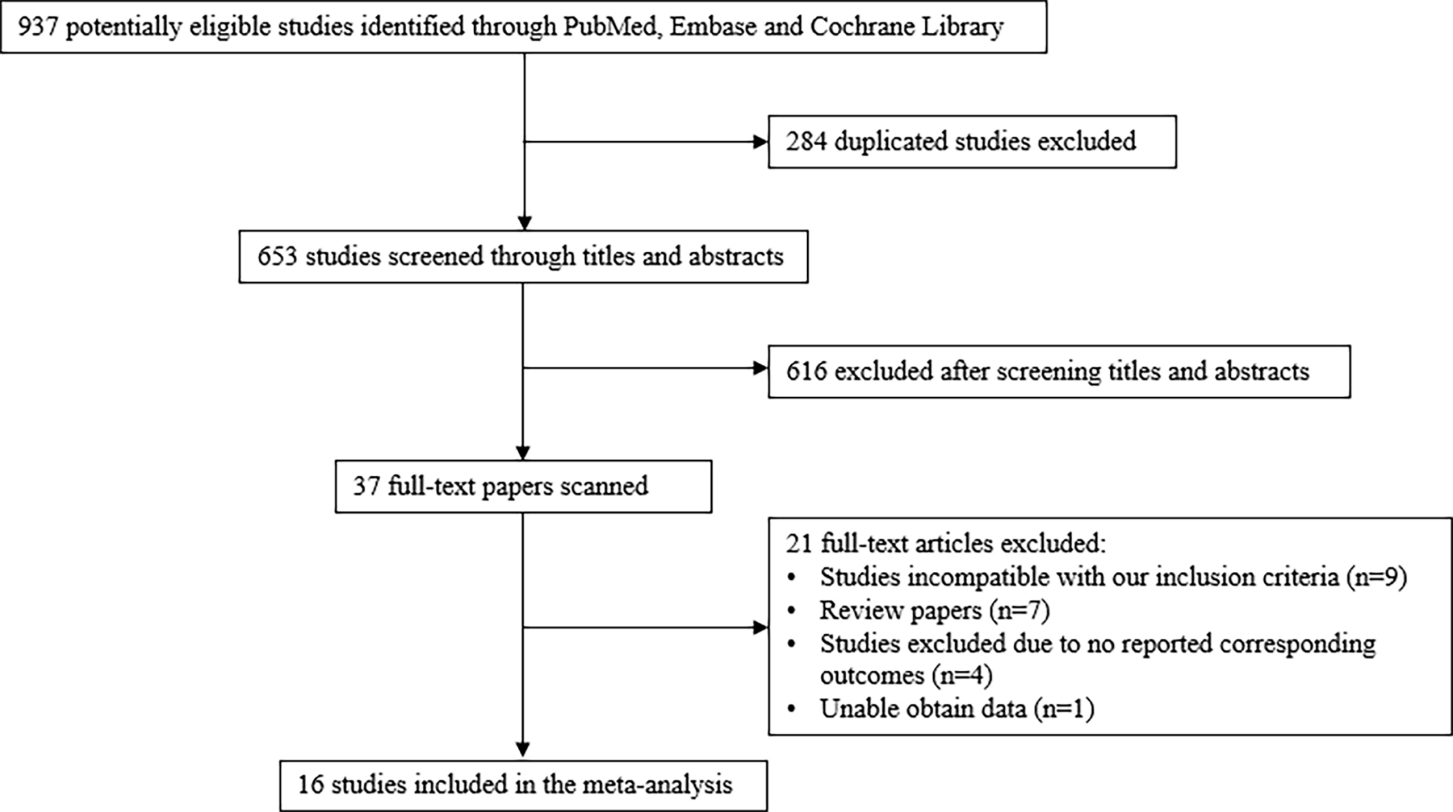
Literature search and screening process.

A total of 653 studies were identified after duplicates removal. After screening titles and abstracts, the full text was retrieved for 37 studies. Of these, 21 articles were excluded: 9 were incompatible with our inclusion criteria, 7 were reviews, 4 did not report corresponding outcomes, and 1 did not provide corresponding data. Finally, a total of 16 studies involving 5,411 patients were selected for the final analysis (**Figure 1**) (9–22, 31, 32).

Among the 16 studies, 16 investigated the outcome differences between the e13a2 and e14a2 groups, and 8 the differences between the three groups (e13a2 vs e14a2 vs both) (9, 12–15, 19, 22, 31). The detailed characteristics of the studies and patients are given in **Table 1**. The methodological quality of the included studies was high (11 of 16) to moderate (5 of 16) according to the NOS (**Table S2**). No significant publication bias was observed for primary outcomes (*P* = 0.539; **Figure S1**).

**Table 1:** Characteristics of the included trials and participants.

Study	study type	Transcript type	Total patients	Type of TKI therapy	Criterion for DMR	Male ratio (%)	Age (median)	Median follow-up (months)	Risk score			Median baseline laboratory values (range)		
									High (%)	Intermediate (%)	Low (%)	Hb (g/dl)	WBC (10^9^/L)	Plt (10^9^/L)
Lucas et al., 2009 (20)	retrospective cohort	e13a2	32	IM	NR	51	50	NR	42^a^	28^a^	30^a^	NR	NR	NR
		e14a2	39											
Hanfstein et al., 2014 (12)	retrospective cohort	e13a2	451	IM	MR^4^	62	52	43	4^b^	NA	96^b^	12 (5-19)	78 (3-630)	420 (34-3020)
		e14a2	496											
		e13a2+e14a2	158											
Jain et al., 2016 (13)	retrospective cohort	e13a2	200	IM	MR^4.5^	NR	49	88	6^a^	24^a^	70^a^	12 (11-14)	30 (12-71)	358 (268-493)
		e14a2	196											
		e13a2+e14a2	85											
Lin et al., 2016 (14)	retrospective cohort	e13a2	61	IM	NR	54	60	NR	NR	NR	NR	NR	NR	NR
		e14a2	83											
		e13a2+e14a2	22											
Castagnetti et al., 2017 (15)	retrospective cohort	e13a2	203	IM	MR^4^	58	52	75	23^a^	37^a^	39^a^	12 (6-18)	52 (1-491)	401 (101-2770)
		e14a2	290											
		e13a2+e14a2	60											
Claudiani et al., 2017 (32)	retrospective cohort	e13a2	27	IM or NIL or DAS	NR	34	51	26	27^a^	29^a^	44^a^	NR	NR	NR
		e14a2	37											
Pagnano et al., 2017 (9)	retrospective cohort	e13a2	56	IM	NR	60	48	80	32^a^	37^a^	31^a^	13 (6-17)	23 (6-234)	334 (139-3363)
		e14a2	94											
		e13a2+e14a2	20											
Pfirrmann et al., 2017 (22)	retrospective cohort	e13a2	565	IM	NR	59	51	78	13^c^	27^c^	60^c^	NR	NR	376 (34–4920)
		e14a2	738											
		e13a2+e14a2	191											
Rostami et al., 2017 (21)	retrospective cohort	e13a2	25	IM	NR	53	49	48	NR	NR	NR	NR	130 (23-550)	383 (168-1547)
		e14a2	35											
D’Adda et al., 2019 (10)	retrospective cohort	e13a2	67	IM or NIL or DAS	MR^4^	49	63	68	22^a^	38^a^	39^a^	NR	NR	NR
		e14a2	106											
Greenfield et al., 2019 (16)	retrospective cohort	e13a2	20	IM	MR^4.5^	61	52	30	6^b^	NA	94^b^	12 (6-16)	141 (5-563)	476 (93-2507)
		e14a2	49											
Sazawal et al., 2019 (17)	retrospective cohort	e13a2	104	IM	NR	64	NR	NR	NR	NR	NR	NR	NR	NR
		e14a2	288											
Genthon et al., 2020 (18)	retrospective cohort	e13a2	51	NIL	MR^4.5^	53	51	49	23^a^	43^a^	33^a^	12 (7-16)	131 (5-623)	358 (83-1999)
		e14a2	63											
Mulas et al., 2020 (19)	retrospective cohort	e13a2	51	NIL	MR^4^	56	50	44	12^a^	33^a^	55^a^	12 (6-17)	71 (2-355)	364 (61-1595)
		e14a2	108											
		e13a2+e14a2	24											
Shanmuganathan et al., 2021 (31)	retrospective cohort	e13a2	43	IM or NIL or DAS	NR	53	61	NR	16^a^	38^a^	46^a^	NR	NR	NR
		e14a2	51											
		e13a2+e14a2	20											
Marce et al., 2021 (11)	retrospective cohort	e13a2	76	IM	MR^4/4.5^	52	56	72	15^a^	41^a^	44^a^	NR	NR	364 (21-2236)
		e14a2	126											

TKI, tyrosine kinase inhibitor; DMR, deep molecular response; NR, not reported; NA, not applicable; IM, imatinib; NIL, nilotinib; DAS, dasatinib; Hb, hemoglobin; WBC, white blood cells; Plt, platelets; a, sokal score; b, EUTOS score; c, EUTOS long-term survival score.

### Major Molecular Response by Transcript Type

**Figure 2:**
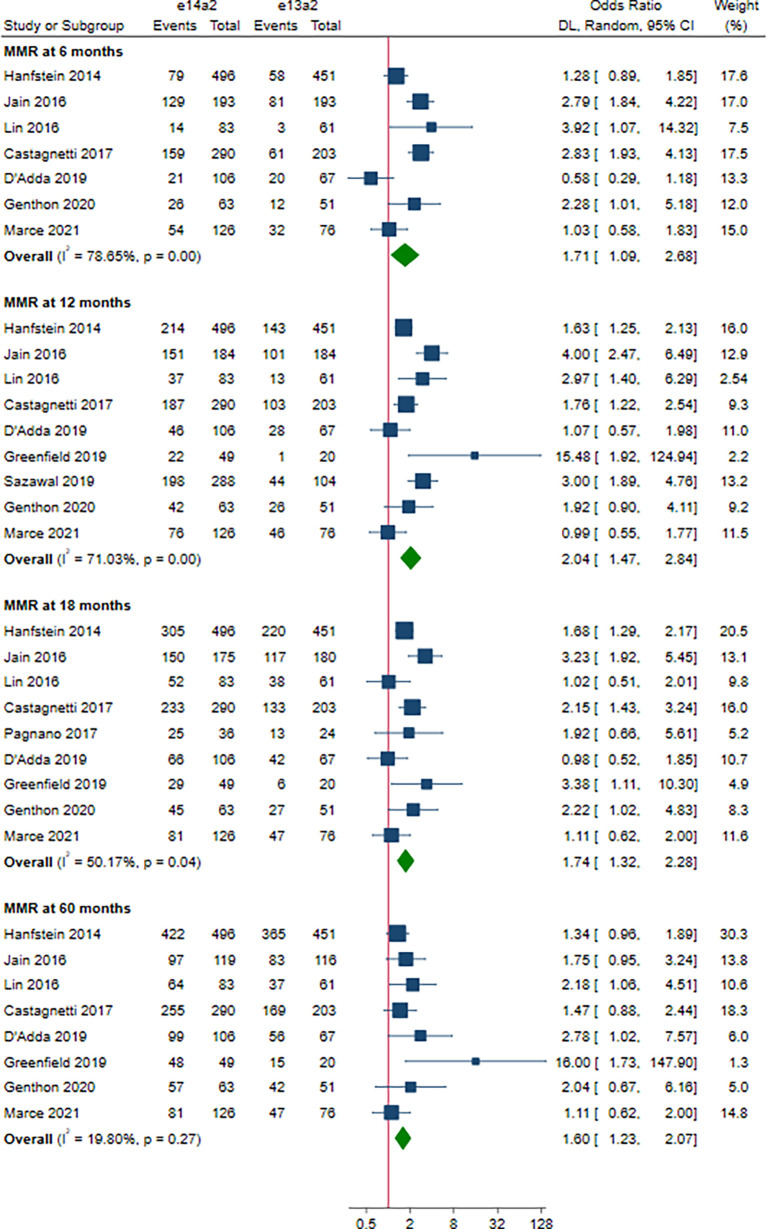
E14a2 versus e13a2: rate of patients who achieved major molecular response at 6, 12, 18 and 60 months.

**Figure 3:**
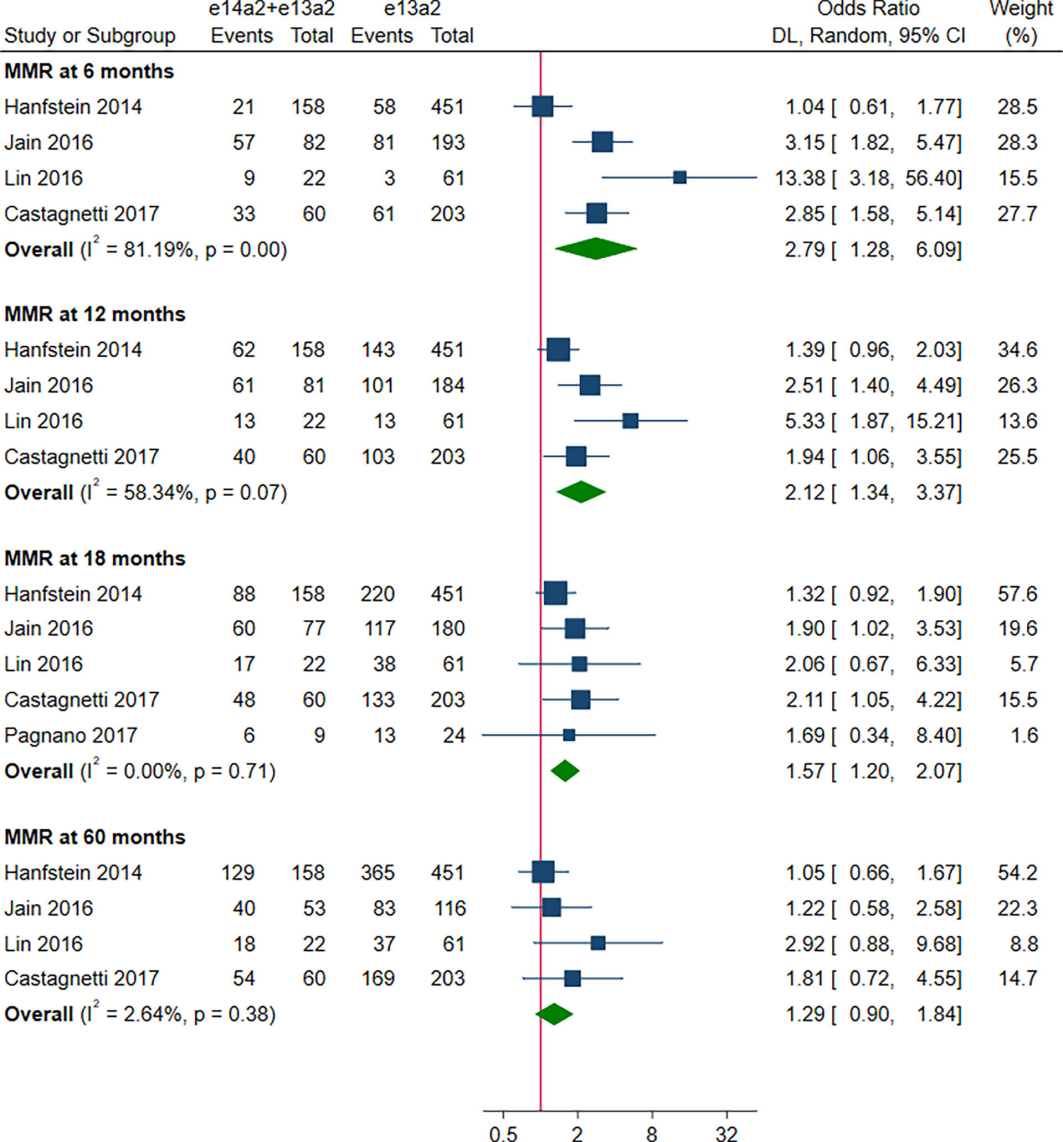
E14a2 + e13a2 versus e13a2: rate of patients who achieved major molecular response at 6, 12, 18 and 60 months.

As shown in **Figure 2**, the rate of MMR was significantly higher in patients with e14a2 transcripts as compared to patients with e13a2 transcripts at 6, 12, 18 and 60 months, resulting in an OR of 1.71 (95% CI: 1.09-2.68, I^2^ = 78.65%, 7 studies), 2.04 (95% CI: 1.47-2.84, I^2^ = 71.03%, 9 studies), 1.74 (95% CI: 1.32-2.28, I^2^ = 50.17%, 9 studies) and 1.60 (95% CI: 1.23-2.07, I^2^ = 19.80%, 8 studies), respectively. Compared to e13a2 transcripts, patients co-expressing e13a2 and e14a2 transcripts achieved higher MMR rates at 6, 12 and 18 months but not at 60 months, with an OR of 2.79 (95% CI: 1.28-6.09, I^2^ = 81.19%, 4 studies), 2.12 (95% CI: 1.34-3.37, I^2^ = 58.34%, 4 studies), 1.57 (95% CI: 1.20-2.07, I^2^ = 0.00%, 5 studies) and 1.29 (95% CI: 0.90-1.84, I^2^ = 2.64%, 4 studies), respectively (**Figure 3**). No significant difference in MMR was found between e14a2 arm and e14a2+e13a2 arm at all-time points (**Figure S2**). According to the subgroup analysis, the effects of different transcripts on MMR at 6, 12, 18 and 60 months were not affected by characteristics of the studies and patients (year of publication, risk of bias, sample size, type of TKI therapy, median follow-up, age, sex, risk score, baseline laboratory values) (data not shown).

### Deep Molecular Response by Transcript Type

**Figure 4:**
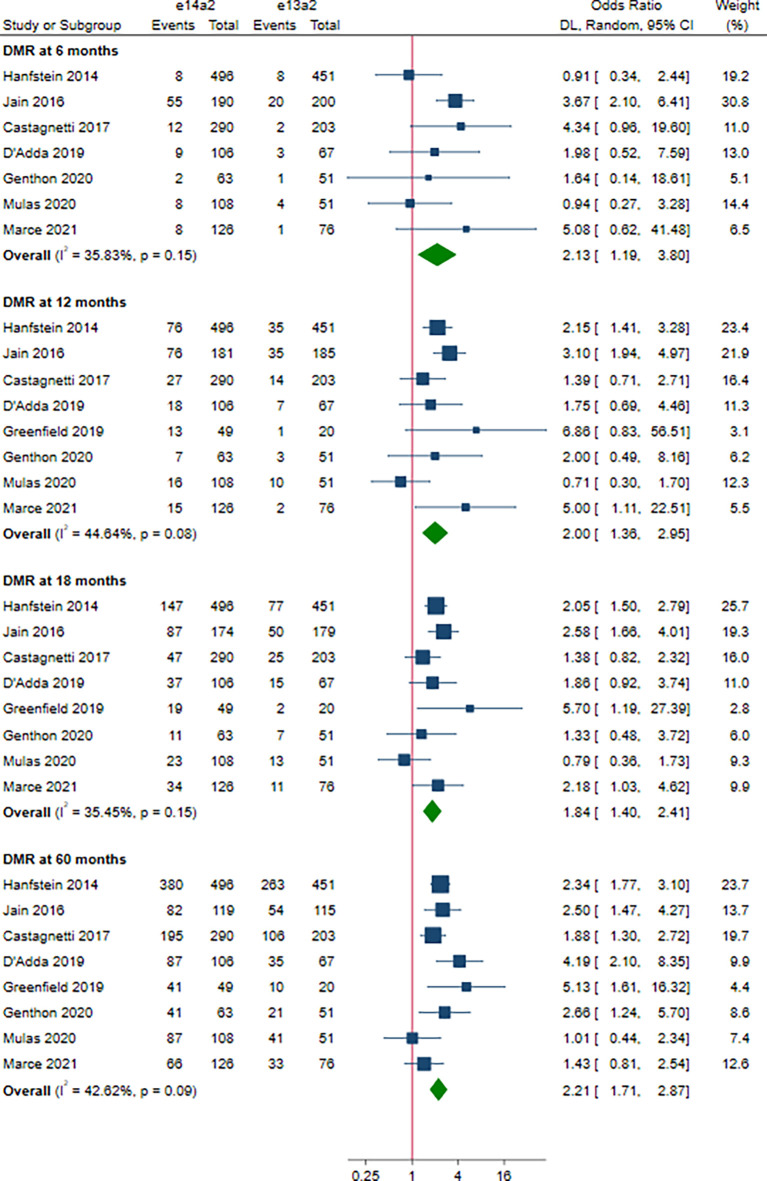
E14a2 versus e13a2: rate of patients who achieved deep molecular response at 6, 12, 18 and 60 months.

**Figure 5:**
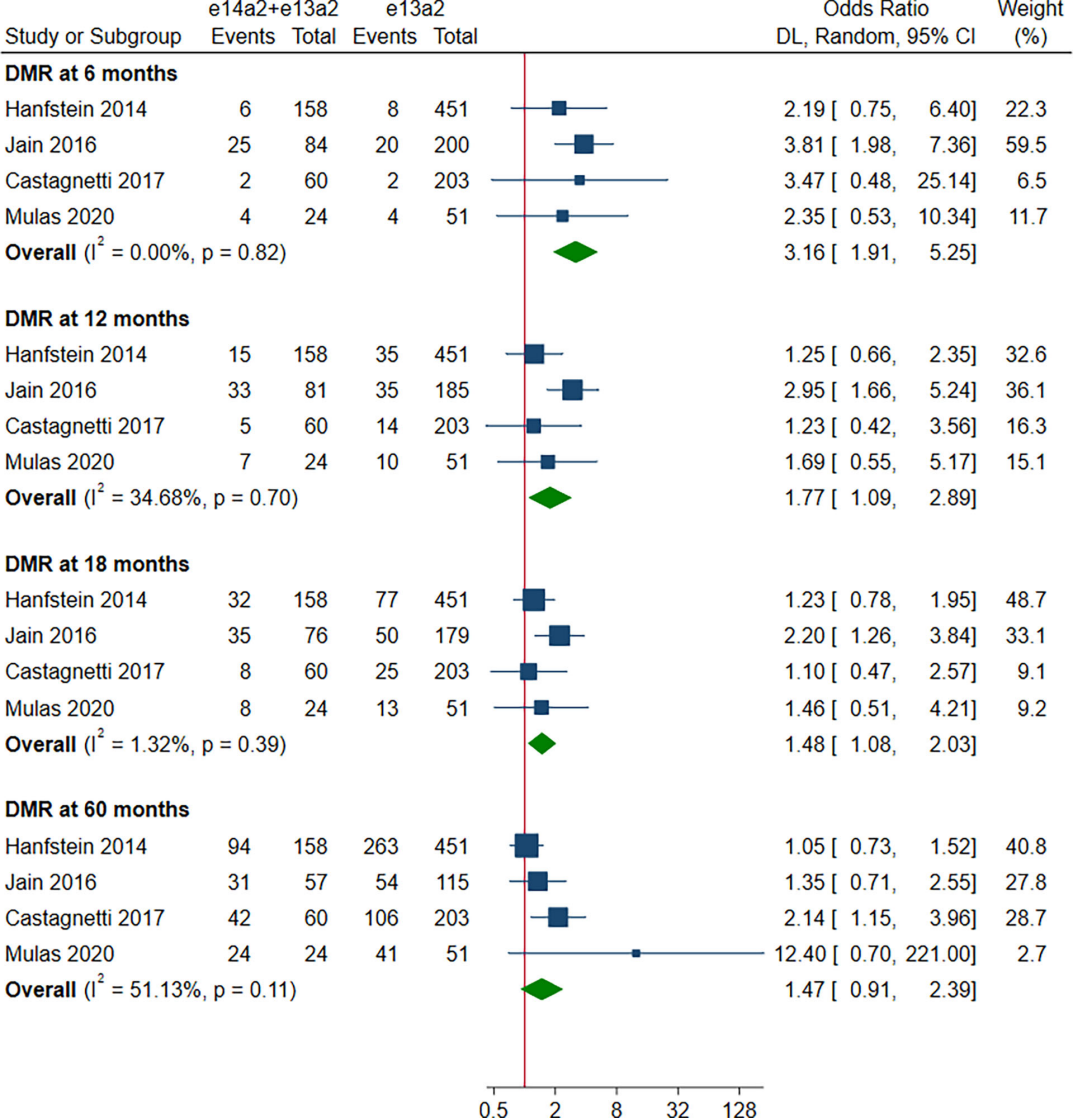
E14a2 + e13a2 versus e13a2: rate of patients who achieved deep molecular response at 6, 12, 18 and 60 months.

A total of 8 studies involving 2,880 patients were included in this part. Of these, DMR is defined as MR^4^ in 4 studies (10, 12, 15, 19), MR^4.5^ in 3 studies (13, 16, 18), and MR^4^/MR^4.5^ in 1 study (11). Compared to e13a2, the patients with e14a2 transcripts had a favorable effect on DMR at 6, 12, 18 and 60 months, with an OR of 2.13 (95% CI: 1.19-3.80, I^2^ = 35.83%, 7 studies), 2.00 (95% CI: 1.36-2.95, I^2^ = 44.64%, 8 studies), 1.84 (95% CI: 1.40-2.41, I^2^ = 35.45%, 8 studies) and 2.21 (95% CI: 1.71-2.87, I^2^ = 42.62%, 8 studies), respectively (**Figure 4**). Given the moderate heterogeneity, meta-regression analyses were performed, considering year of publication, criterion for DMR, risk of bias, sample size, type of TKI therapy, median follow-up, age, sex, risk score, baseline laboratory values, but the above variables could not explain the heterogeneity (**Table S3**). DMR rates were significantly higher in patients co-expressing both transcripts as compared to patients expressing only e13a2 transcript alone at 6, 12 and 18 months but not at 60 months, with an OR of 3.16 (95% CI: 1.91-5.25, I^2^ = 0.00%, 4 studies), 1.77 (95% CI: 1.09-2.89, I^2^ = 34.68%, 4 studies), 1.48 (95% CI: 1.08-2.03, I^2^ = 1.32%, 4 studies) and 1.47 (95% CI: 0.91-2.39, I^2 =^ 51.13%, 4 studies), respectively (**Figure 5**). No significant difference in DMR was found between e14a2 arm and e14a2+e13a2 arm at all-time points (**Figure S3**). The subgroup analysis did not show any significant differences (data not shown).

### Survival Outcomes According to Transcript Type

**Figure 6:**
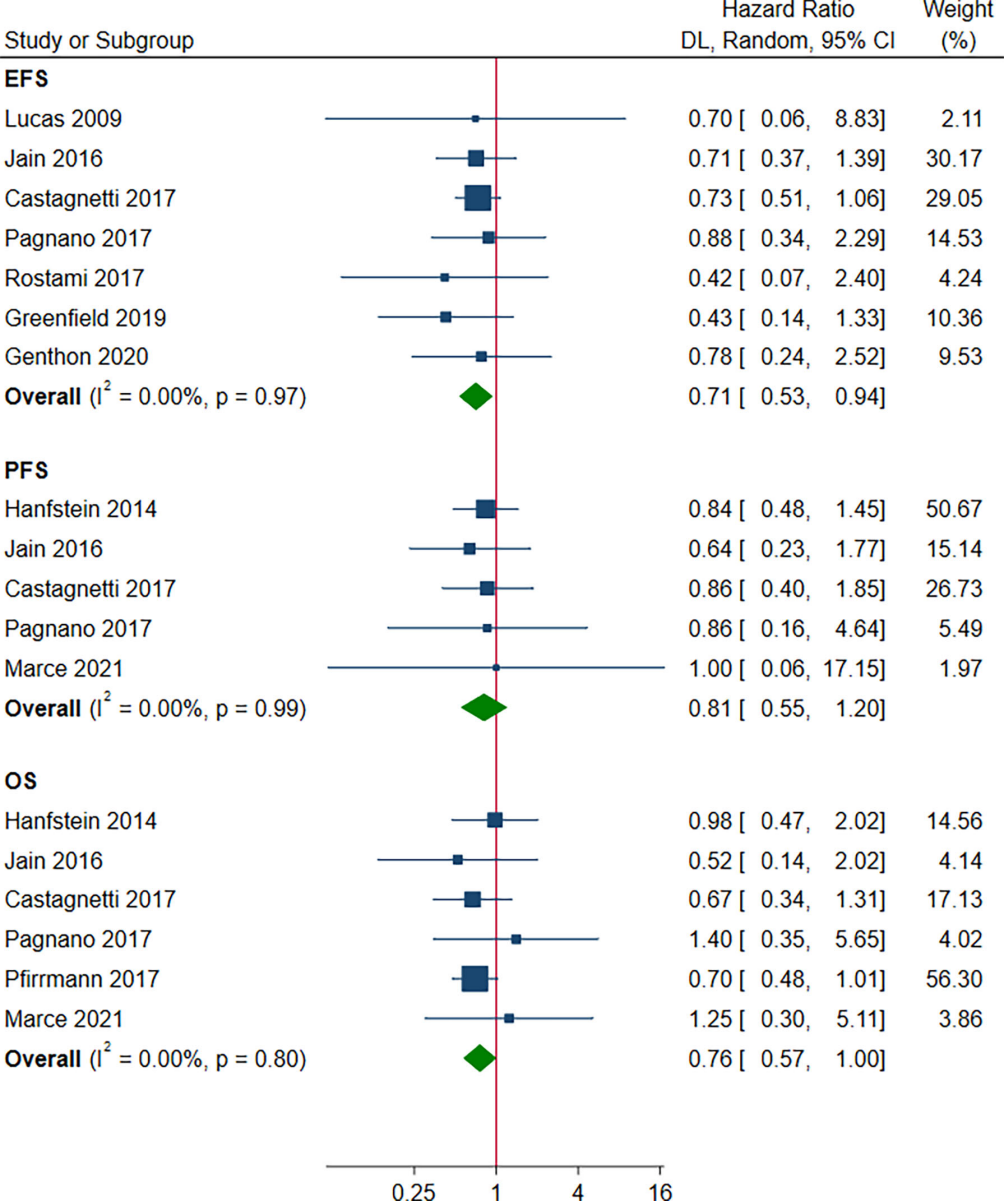
Survival outcomes for patients with the e14a2 and e13a2 transcripts.

There was a statistically significant improvement in favor of the patients with e14a2 transcripts compared to patients with e13a2 transcripts in terms of EFS and OS (HR 0.71, 95% CI 0.53-0.94, I^2^ = 0.00%, 7 studies; HR 0.76, 95% CI 0.57-1.00, I^2^ = 0.00%, 6 studies, **Figure 6**). However, no differences between the other groups were observed with regard to EFS, PFS and OS (**Figures S4, S5**).

### Treatment-Free Remission According to Transcript Type

**Figure 7:**
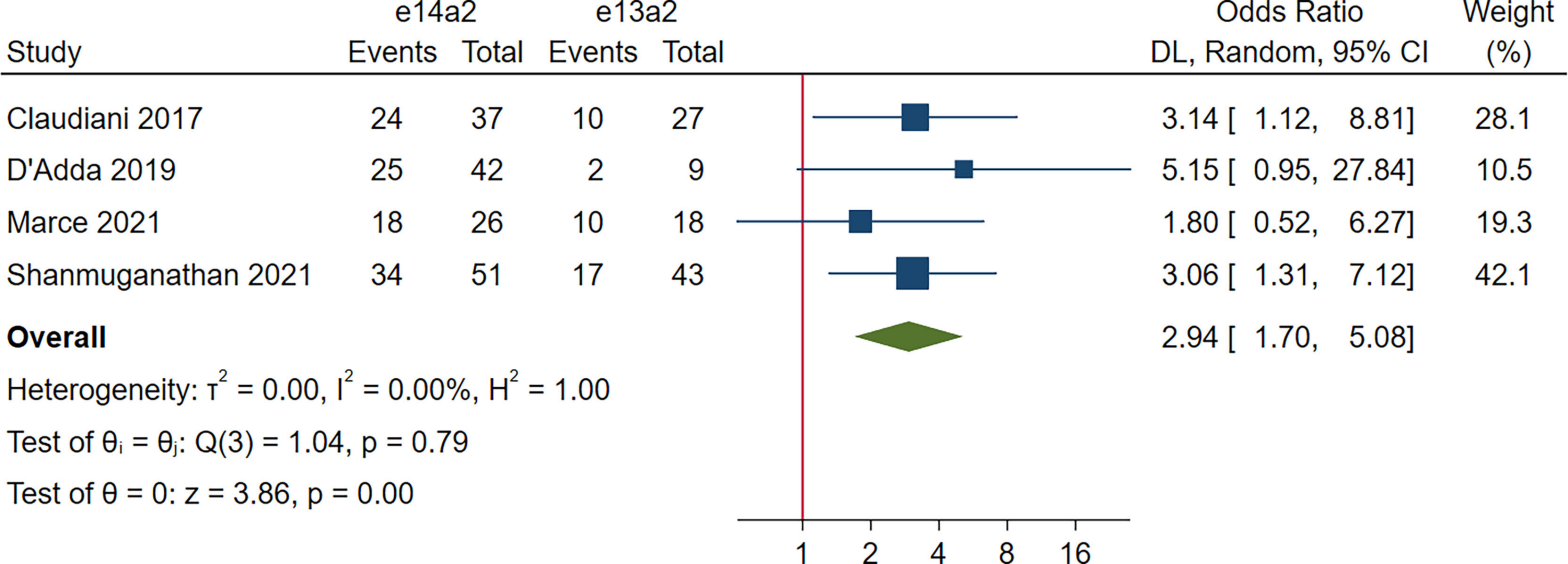
Rate of treatment-free remission for patients with the e14a2 and e13a2 transcripts.

Four studies, 253 patients, were included in this analysis. The rate of TFR was significantly improved in patients with e14a2 transcripts compared to those with e13a2 transcripts (OR 2.94, 95% CI 1.70-5.08, I^2^ = 0.00%; **Figure 7**).

### Network Meta-Analysis

Network diagrams were presented in **Figure S6**. Comparing results from NMA and traditional pairwise meta-analysis suggested that direct and indirect evidence was roughly consistent (**Figures S7–S9**). Based on the SUCRA analyses, the e14a2 transcript ranked first with 77.3%, 79.0%, 72.2%, 84.8% and 93.3% probabilities of providing the highest MMR at 60 months, DMR at 60 months, EFS, PFS and OS, respectively (**Table 2**).

**Table 2:** SUCRA results of evaluated outcomes for each transcript type.

Transcript types	MMR			DMR			EFS	PFS	OS
	12 months	18 months	60 months	12 months	18 months	60 months			
e13a2	0	0	0	0.1%	0	0.1%	0.4%	8.7%	6.2%
e14a2	52.0%	66.6%	77.3%	67.8%	85.4%	79.0%	72.2%	84.8%	93.3%
e13a2+e14a2	48.0%	33.4%	22.7%	32.1%	14.5%	20.9%	27.4%	6.5%	0.5%

SUCRA, surface under the cumulative ranking.

## Discussion

To our knowledge, this is the first meta-analysis aiming to evaluate the impact of BCR-ABL transcript type on outcome in TKI-treated patients with CML. To fully leverage available data and increase confidence in our results, several analytical approaches were used including traditional meta-analysis and network meta-analysis. Compared with e13a2 transcripts, there was a statistically significant advantage for patients with e14a2 (alone or with co-expressed e13a2) in terms of MMR and DMR at 6, 12 and 18 months. This benefit was sustained up to 5 years for patients with e14a2 transcripts, but not for patients with both transcripts. The expression of e14a2 also improved probability of EFS and OS throughout treatment period. Importantly, having e14a2 transcripts were associated with a higher rate of TFR in CML patients attempting TKI discontinuation. By network meta-analysis, results were similar, confirming the robustness of the results. Rank analyses showed that the e14a2 transcript had the highest probability to be the most favorable transcript type for the majority of outcomes, followed by both and e13a2.

As evidence continues to emerge from TFR studies, discontinuation of TKI therapy is feasible in the most of patients with sufficient TKI response. However, the eligibility criteria for patients maintaining optimal TKI discontinuation is rather strict (i.e., a minimum of 5 years of TKI therapy and (> 3 years of sustained MR^4^ or > 2 years of sustained MR^4.5^)) (25). Our meta-analysis indicated that the DMR at 60 months was still higher in the e14a2 arm (OR 2.21, 95% CI: 1.71-2.87), suggesting 121% more patients expressing e14a2 transcripts qualified for entering TFR phase compared to those expressing e13a2 transcripts. So far, well-designed studies to directly investigate the correlation between different transcripts and the maintenance of TFR have less frequently been performed. Claudiani et al. (32) and Shanmuganathan et al. (31) found that probability of TFR was higher for patients expressing e14a2 transcripts, whereas D’Adda et al. (10) and Marce et al. (11) demonstrated that transcript types did not affect TFR rates. Our exploratory meta-analysis showed a 217% increase in the rate of TFR maintenance in e14a2 patients attempting TKI discontinuation. In addition, previous studies have demonstrated several factors contributing to successful TFR with duration of DMR appearing to be the most important predictor (33–36). Thus, taken together, it is suggested that e14a2 transcripts could serve as a strong parameter allowing for successful TFR. Of note, due to the lack of power stemming from the limited number of studies included and the small sample size, the result concerning correlation between the transcript type and TFR maintenance rate may not be robust (only 156 and 97 patients in the e14a2 and the e13a2 arms, respectively, were pooled). Taking important values of TFR in treatment of CML into consideration, further studies in larger patient cohorts are required to demonstrate this correlation.

Complete cytogenetic response (CCyR) results were not calculated, since they are insufficiently sensitive to monitor response. On the other hand, CCyR is also not optimal in our study comparing differences between typical BCR-ABL1 transcripts. The up-dated 2020 ELN guidelines recommend the BCR-ABL1 transcript levels at specific time points as the monitoring milestones for treating CML and achieving MMR from 12 months onward is regarded as the optimal response (25). As far as MMR rates are concerned, our results showed superiority which is sustained over 60 months in the e14a2 arm compared to the e13a2 arm. Indeed, patients who maintain MMR throughout long-term follow-up are not likely to progress, but rather show good clinical results. So far, e14a2 has not yet been evaluated and included in prognostic systems, i.e., Sokal, Euro, EUTOS and ELTS (37–40). For high-and intermediate-risk patients stratified by current risk scores, the physicians are more likely to use new-generation TKIs for treating them in case of treatment failure or in order to achieve TFR. However, since the increased number of CML-unrelated deaths occurred in CML patients who are still in remission, the physicians making clinical decisions also consider the patient’s characteristics, comorbidity and the distinct toxic effect profile of the different TKIs. For example, pleuro-pulmonary disease and arteriovascular disease are strong contraindications to dasatinib and nilotinib, respectively (41–43). In many situations, the benefit versus risk is difficult to balance. Our results provided a possibility that patients with e14a2 transcripts could reduce risk stratification to a certain extent, thereby receiving effective imatinib therapy with relatively less toxicity. In addition, Jain et al. (13) even found that the rates of MMR for patients with e14a2 transcripts treated with imatinib were similar to that of patients treated with second-generation TKIs. Thus, to obtain more accurate benefit-risk profile, we strongly suggest incorporating the e14a2 transcript into the prognostic system and giving it appropriate weight.

Although EFS and OS in each study were no statistical difference, the pooled results resulted significant (**Figure 6**). There may be several reasons for this contrary observation. First, statistical heterogeneity was mild (I^2^ = 0.00%) and the point estimates (rectangles) from almost all studies were located on the left of the vertical line. Second, life expectancy of patients with typical BCR-ABL1 transcripts treated with all TKIs is close to that of the general population. Also, progression to AP/BC and CML-related mortality are rarely encountered after 12 months of TKI therapy (44). If differences in long-term survival outcomes exist, less statistical power owing to fewer events would need a large patient sample to discover such differences. Our meta-analysis increased the sample size and reduced the widths of CI, therefore providing statistically significant results.

In our meta-analysis, the advantage of the e13a2 and e14a2 co-expression was demonstrated within 18 months of treatment in terms of both MMR and DMR. Even so, no significant difference was found in evaluated outcomes at 60 months as compared to e13a2, neither for molecular response nor for survival. This phenomenon is reasonable because e13a2 cells are more persistent and the prognosis of patients with e13a2 is worse (45). Thus, we suppose that patients with co-expression of both transcripts after experiencing long-term TKI treatment would probably require more careful molecular monitoring or derive more benefit from switching to new-generation TKIs.

Our meta-analysis has several limitations: 1) The definition of DMR were not uniform such that DMR was defined as MR^4^ in some studies and as MR^4.5^ in others, potentially reducing precision. 2) As with any meta-analysis, our study lacked individual data that might have provided additional details such as sustained DMR which was more critical for patients attempting TKI discontinuation. 3) Studies were pooled with different characteristics of the patients and designs such as TKI type, median follow-up and amplification efficiency. Despite this limitation, heterogeneity was mild for the primary outcomes across these studies; we also minimized the influence of heterogeneity by using a random-effects model, especially for the secondary outcomes with severe heterogeneity. Additionally, subgroup analyses were performed according to characteristics of the patients and designs, and these provided concordant results. 4) For some outcomes, the number of studies included was limited, which could increase uncertainty of the results. 5) Our meta-analysis might have been limited by the retrospective nature of the included studies.

## Conclusions

In conclusion, the expression of e14a2 is related to a faster, deeper and more sustained molecular response compared to e13a2. This superiority in response translates in improved long-term EFS and OS. From 12 months onward, having the e14a2 transcript ranked first to achieve all outcomes. Importantly, despite relatively small numbers, the expression of e14a2 may have a positive impact on TFR. Our meta-analysis shows that the e14a2 transcript can be added to the list of prognostic factors to guide clinical decisions in treating CML, especially at a time when TFR is becoming the ultimate goal of therapy.

## Data Availability Statement

The original contributions presented in the study are included in the article/**Supplementary Material**. Further inquiries can be directed to the corresponding authors.

## Author Contributions

KC, FC, and QC contributed to the conception and design this study. WH and JL carried out the development of the methodology. NX, KT, and PX analyzed and interpreted the data. KC and YR wrote the manuscript and approved the final submission of the study. All authors read and approved the final manuscript.

## Conflict of Interest

The authors declare that the research was conducted in the absence of any commercial or financial relationships that could be construed as a potential conflict of interest.

## Publisher’s Note

All claims expressed in this article are solely those of the authors and do not necessarily represent those of their affiliated organizations, or those of the publisher, the editors and the reviewers. Any product that may be evaluated in this article, or claim that may be made by its manufacturer, is not guaranteed or endorsed by the publisher.

## References

[1] Ren R . Mechanisms of BCR-ABL in the Pathogenesis of Chronic Myelogenous Leukaemia. Nat Rev Cancer (2005) 5:172–83. doi: 10.1038/nrc1567 15719031

[2] Groffen J Stephenson JR Heisterkamp N de Klein A Bartram CR Grosveld G . Philadelphia Chromosomal Breakpoints are Clustered Within a Limited Region, Bcr, on Chromosome 22. Cell (1984) 36:93–9. doi: 10.1016/0092-8674(84)90077-1 6319012

[3] Melo JV . The Diversity of BCR-ABL Fusion Proteins and Their Relationship to Leukemia Phenotype. Blood (1996) 88:2375–84. doi: 10.1182/blood.V88.7.2375.bloodjournal8872375 8839828

[4] Shah NP . Front-Line Treatment Options for Chronic-Phase Chronic Myeloid Leukemia. J Clin Oncol (2018) 36:220–4. doi: 10.1200/JCO.2017.75.4663 29206554

[5] Brunner AM Campigotto F Sadrzadeh H Drapkin BJ Chen YB Neuberg DS . Trends in All-Cause Mortality Among Patients With Chronic Myeloid Leukemia: A Surveillance, Epidemiology, and End Results Database Analysis. Cancer (2013) 119:2620–9. doi: 10.1002/cncr.28106 23625575

[6] Cortes JE Saglio G Kantarjian HM Baccarani M Mayer J Boque C . Final 5-Year Study Results of DASISION: The Dasatinib Versus Imatinib Study in Treatment-Naive Chronic Myeloid Leukemia Patients Trial. J Clin Oncol (2016) 34:2333–40. doi: 10.1200/JCO.2015.64.8899 PMC5118045 27217448

[7] Hochhaus A Saglio G Hughes TP Larson RA Kim DW Issaragrisil S . Long-Term Benefits and Risks of Frontline Nilotinib vs Imatinib for Chronic Myeloid Leukemia in Chronic Phase: 5-Year Update of the Randomized ENESTnd Trial. Leukemia (2016) 30:1044–54. doi: 10.1038/leu.2016.5 PMC4858585 26837842

[8] Baccarani M Rosti G Soverini S . Chronic Myeloid Leukemia: The Concepts of Resistance and Persistence and the Relationship With the BCR-ABL1 Transcript Type. Leukemia (2019) 33:2358–64. doi: 10.1038/s41375-019-0562-1 31455852

[9] Pagnano KBB Miranda EC Delamain MT Duarte GO de Paula EV Lorand-Metze I . Influence of BCR-ABL Transcript Type on Outcome in Patients With Chronic-Phase Chronic Myeloid Leukemia Treated With Imatinib. Clin Lymphoma Myeloma Leuk (2017) 17:728–33. doi: 10.1016/j.clml.2017.06.009 28822797

[10] D'Adda M Farina M Schieppati F Borlenghi E Bottelli C Cerqui E . The E13a2 BCR-ABL Transcript Negatively Affects Sustained Deep Molecular Response and the Achievement of Treatment-Free Remission in Patients With Chronic Myeloid Leukemia Who Receive Tyrosine Kinase Inhibitors. Cancer (2019) 125(10):1674–82. doi: 10.1002/cncr.31977 30707758

[11] Marce S Xicoy B Garcia O Cabezon M Estrada N Velez P . Impact of BCR-ABL1 Transcript Type on Response, Treatment-Free Remission Rate and Survival in Chronic Myeloid Leukemia Patients Treated With Imatinib. J Clin Med (2021) 10(14):3146. doi: 10.3390/jcm10143146 34300312 PMC8307111

[12] Hanfstein B Lauseker M Hehlmann R Saussele S Erben P Dietz C . Distinct Characteristics of E13a2 Versus E14a2 BCR-ABL1 Driven Chronic Myeloid Leukemia Under First-Line Therapy With Imatinib. Haematologica (2014) 99:1441–7. doi: 10.3324/haematol.2013.096537 PMC4562532 24837466

[13] Jain P Kantarjian H Patel KP Gonzalez GN Luthra R Kanagal Shamanna R . Impact of BCR-ABL Transcript Type on Outcome in Patients With Chronic-Phase CML Treated With Tyrosine Kinase Inhibitors. Blood (2016) 127:1269–75. doi: 10.1182/blood-2015-10-674242 PMC4786836 26729897

[14] Lin HX Sjaarda J Dyck J Stringer R Hillis C Harvey M . Gender and BCR-ABL Transcript Type are Correlated With Molecular Response to Imatinib Treatment in Patients With Chronic Myeloid Leukemia. Eur J Haematol (2016) 96:360–6. doi: 10.1111/ejh.12597 26059983

[15] Castagnetti F Gugliotta G Breccia M Iurlo A Levato L Albano F . The BCR-ABL1 Transcript Type Influences Response and Outcome in Philadelphia Chromosome-Positive Chronic Myeloid Leukemia Patients Treated Frontline With Imatinib. Am J Hematol (2017) 92:797–805. doi: 10.1002/ajh.24774 28466557

[16] Greenfield G McMullan R Robson N McGimpsey J Catherwood M McMullin MF . Response to Imatinib Therapy is Inferior for E13a2 BCR-ABL1 Transcript Type in Comparison to E14a2 Transcript Type in Chronic Myeloid Leukaemia. BMC Hematol (2019) 19:7. doi: 10.1186/s12878-019-0139-2 31073408 PMC6498698

[17] Sazawal S Chhikara S Singh K Chaubey R Mahapatra M Seth T . Distribution of Common BCR-ABL Fusion Transcripts and Their Impact on Treatment Response in Imatinib Treated CML Patients: A Study From India. Indian J Pathol Microbiol (2019) 62:256–60. doi: 10.4103/IJPM.IJPM_726_17 30971550

[18] Genthon A Nicolini FE Huguet F Colin-Gil C Berger M Saugues S . Influence of Major BCR-ABL1 Transcript Subtype on Outcome in Patients With Chronic Myeloid Leukemia in Chronic Phase Treated Frontline With Nilotinib. Oncotarget (2020) 11:2560–70. doi: 10.18632/oncotarget.27652 PMC7335668 32655840

[19] Mulas O Caocci G Annunziata M Martino B Luciano L Castagnetti F . Favorable Outcome of Chronic Myeloid Leukemia Co-Expressing E13a2 and E14a2 Transcripts, Treated With Nilotinib. Hematol Oncol (2020) 38:607–10. doi: 10.1002/hon.2765 32602167

[20] Lucas CM Harris RJ Giannoudis A Davies A Knight K Watmough SJ . Chronic Myeloid Leukemia Patients With the E13a2 BCR-ABL Fusion Transcript Have Inferior Responses to Imatinib Compared to Patients With the E14a2 Transcript. Haematologica (2009) 94:1362–7. doi: 10.3324/haematol.2009.009134 PMC2754951 19713230

[21] Rostami G Hamid M Jalaeikhoo H . Impact of the BCR-ABL1 Fusion Transcripts on Different Responses to Imatinib and Disease Recurrence in Iranian Patients With Chronic Myeloid Leukemia. Gene (2017) 627:202–6. doi: 10.1016/j.gene.2017.06.018 28627443

[22] Pfirrmann M Evtimova D Saussele S Castagnetti F Cervantes F Janssen J . No Influence of BCR-ABL1 Transcript Types E13a2 and E14a2 on Long-Term Survival: Results in 1494 Patients With Chronic Myeloid Leukemia Treated With Imatinib. J Cancer Res Clin Oncol (2017) 143:843–50. doi: 10.1007/s00432-016-2321-2 PMC11818951 28083711

[23] Liberati A Altman DG Tetzlaff J Mulrow C Gotzsche PC Ioannidis JP . The PRISMA Statement for Reporting Systematic Reviews and Meta-Analyses of Studies That Evaluate Health Care Interventions: Explanation and Elaboration. J Clin Epidemiol (2009) 62:e1–34. doi: 10.1016/j.jclinepi.2009.06.006 19631507

[24] Ercaliskan A Eskazan AE . The Impact of BCR-ABL1 Transcript Type on Tyrosine Kinase Inhibitor Responses and Outcomes in Patients With Chronic Myeloid Leukemia. Cancer (2018) 124:3806–18. doi: 10.1002/cncr.31408 29694669

[25] Hochhaus A Baccarani M Silver RT Schiffer C Apperley JF Cervantes F . European LeukemiaNet 2020 Recommendations for Treating Chronic Myeloid Leukemia. Leukemia (2020) 34:966–84. doi: 10.1038/s41375-020-0776-2 PMC7214240 32127639

[26] Tierney JF Stewart LA Ghersi D Burdett S Sydes MR . Practical Methods for Incorporating Summary Time-to-Event Data Into Meta-Analysis. Trials (2007) 8:16. doi: 10.1186/1745-6215-8-16 17555582 PMC1920534

[27] Lin L Chu H . Quantifying Publication Bias in Meta-Analysis. Biometrics (2018) 74:785–94. doi: 10.1111/biom.12817 PMC5953768 29141096

[28] Tonin FS Rotta I Mendes AM Pontarolo R . Network Meta-Analysis: A Technique to Gather Evidence From Direct and Indirect Comparisons. Pharm Pract (2017) 15:943. doi: 10.18549/PharmPract.2017.01.943 PMC5386629 28503228

[29] Dias S Sutton AJ Ades AE Welton NJ . Evidence Synthesis for Decision Making 2: A Generalized Linear Modeling Framework for Pairwise and Network Meta-Analysis of Randomized Controlled Trials. Med Decision Making An Int J Soc Med Decision Making (2013) 33:607–17. doi: 10.1177/0272989X12458724 PMC3704203 23104435

[30] Lu G Ades AE . Combination of Direct and Indirect Evidence in Mixed Treatment Comparisons. Stat Med (2004) 23:3105–24. doi: 10.1002/sim.1875 15449338

[31] Shanmuganathan N Pagani IS Ross DM Park S Yong ASM Braley JA . Early BCR-ABL1 Kinetics are Predictive of Subsequent Achievement of Treatment-Free Remission in Chronic Myeloid Leukemia. Blood (2021) 137:1196–207. doi: 10.1182/blood.2020005514 32871588

[32] Claudiani S Apperley JF Gale RP Clark R Szydlo R Deplano S . E14a2 BCR-ABL1 Transcript is Associated With a Higher Rate of Treatment-Free Remission in Individuals With Chronic Myeloid Leukemia After Stopping Tyrosine Kinase Inhibitor Therapy. Haematologica (2017) 102:e297–9. doi: 10.3324/haematol.2017.168740 PMC5541883 28495914

[33] Chen KK Du TF Xiong PS Fan GH Yang W . Discontinuation of Tyrosine Kinase Inhibitors in Chronic Myeloid Leukemia With Losing Major Molecular Response as a Definition for Molecular Relapse: A Systematic Review and Meta-Analysis. Front Oncol (2019) 9:372. doi: 10.3389/fonc.2019.00372 31139566 PMC6527744

[34] Ross DM Masszi T Gomez Casares MT Hellmann A Stentoft J Conneally E . Durable Treatment-Free Remission in Patients With Chronic Myeloid Leukemia in Chronic Phase Following Frontline Nilotinib: 96-Week Update of the ENESTfreedom Study. J Cancer Res Clin Oncol (2018) 144(5):945–54. doi: 10.1007/s00432-018-2604-x PMC5916993 29468438

[35] Saussele S Richter J Guilhot J Gruber FX Hjorth-Hansen H Almeida A . Discontinuation of Tyrosine Kinase Inhibitor Therapy in Chronic Myeloid Leukaemia (EURO-SKI): A Prespecified Interim Analysis of a Prospective, Multicentre, non-Randomised, Trial. Lancet Oncol (2018) 19:747–57. doi: 10.1016/S1470-2045(18)30192-X 29735299

[36] Branford S Wang P Yeung DT Thomson D Purins A Wadham C . Integrative Genomic Analysis Reveals Cancer-Associated Mutations at Diagnosis of CML in Patients With High-Risk Disease. Blood (2018) 132:948–61. doi: 10.1182/blood-2018-02-832253 29967129

[37] Hasford J Pfirrmann M Hehlmann R Allan NC Baccarani M Kluin-Nelemans JC . A New Prognostic Score for Survival of Patients With Chronic Myeloid Leukemia Treated With Interferon Alfa. Writing Committee for the Collaborative CML Prognostic Factors Project Group. J Natl Cancer Inst (1998) 90:850–8. doi: 10.1093/jnci/90.11.850 9625174

[38] Hasford J Baccarani M Hoffmann V Guilhot J Saussele S Rosti G . Predicting Complete Cytogenetic Response and Subsequent Progression-Free Survival in 2060 Patients With CML on Imatinib Treatment: The EUTOS Score. Blood (2011) 118:686–92. doi: 10.1182/blood-2010-12-319038 21536864

[39] De Braekeleer M . BCR-ABL1 B3a2 and B2a2 Transcripts in Chronic Myeloid Leukemia: Does it Matter? Eur J Haematol (2016) 96:329–30. doi: 10.1111/ejh.12639 26208040

[40] Geelen IGP Sandin F Thielen N Janssen J Hoogendoorn M Visser O . Validation of the EUTOS Long-Term Survival Score in a Recent Independent Cohort of "Real World" CML Patients. Leukemia (2018) 32:2299–303. doi: 10.1038/s41375-018-0136-7 29743721

[41] Douxfils J Haguet H Mullier F Chatelain C Graux C Dogne JM . Association Between BCR-ABL Tyrosine Kinase Inhibitors for Chronic Myeloid Leukemia and Cardiovascular Events, Major Molecular Response, and Overall Survival: A Systematic Review and Meta-Analysis. JAMA Oncol (2016) 2(5):625–32. doi: 10.1001/jamaoncol.2015.5932 26847662

[42] Jain P Kantarjian H Boddu PC Nogueras-González GM Verstovsek S Garcia-Manero G . Analysis of Cardiovascular and Arteriothrombotic Adverse Events in Chronic-Phase CML Patients After Frontline TKIs. Blood Adv (2019) 3:851–61. doi: 10.1182/bloodadvances.2018025874 PMC6436011 30885996

[43] Hughes TP Laneuville P Rousselot P Snyder DS Rea D Shah NP . Incidence, Outcomes, and Risk Factors of Pleural Effusion in Patients Receiving Dasatinib Therapy for Philadelphia Chromosome-Positive Leukemia. Haematologica (2019) 104:93–101. doi: 10.3324/haematol.2018.188987 30093398 PMC6312029

[44] Sasaki K Kantarjian HM Jain P Jabbour EJ Ravandi F Konopleva M . Conditional Survival in Patients With Chronic Myeloid Leukemia in Chronic Phase in the Era of Tyrosine Kinase Inhibitors. Cancer (2016) 122:238–48. doi: 10.1002/cncr.29745 PMC4707980 26479889

[45] Baccarani M Castagnetti F Gugliotta G Rosti G Soverini S Albeer A . The Proportion of Different BCR-ABL1 Transcript Types in Chronic Myeloid Leukemia. An International Overview. Leukemia (2019) 33(5):1173–83. doi: 10.1038/s41375-018-0341-4 30675008

