# Supplementary material for: Impact of BCR-ABL1 Transcript Type on Outcome in Chronic Myeloid Leukemia Patients Treated With Tyrosine Kinase Inhibitors: A Pairwise and Bayesian Network Meta-Analysis
Source: Front Oncol. 2022 Feb 10;12:841546. doi: 10.3389/fonc.2022.841546 (PMC8867088; doi:10.3389/fonc.2022.841546)

**Supplemental Files**

**Table S1** Search Strategy and Search Results

| **Table S1** Search Strategy and Search Results | | |
| --- | --- | --- |
| **A** PubMed Search | | |
| Entry | PubMed Search Strategy | Results |
| 1 | "Leukemia, Myelogenous, Chronic, BCR-ABL Positive"[Mesh] | 20,806 |
| 2 | (b3a2) OR (b2a2) OR (e14a2) OR (e13a2) | 479 |
| 3 | #1 AND #2 | 304 |
| **B** Embase Search | | |
| Entry | Embase Search Strategy | Results |
| 1 | 'Chronic Myeloid Leukemia'/exp | 48,382 |
| 2 | e13a2 OR e14a2 OR b2a2 OR b3a2 | 875 |
| 3 | #1 AND #2 | 629 |
| **C** Cochrane Library Search | | |
| Entry | Cochrane Library Search Strategy | Results |
| 1 | [ Leukemia, Myelogenous, Chronic, BCR-ABL Positive] explode all trees | 509 |
| 2 | (b3a2) OR (b2a2) OR (e14a2) OR (e13a2) | 34 |
| 3 | #1 AND #2 | 4 |

**Table S2**. Newcastle-Ottawa Scale for assessing the quality of comparative studies in the meta-analysis

| **Study** | Representativeness of the exposed cohort | Selection of the non-exposed cohort | Ascertainment of exposure | Demonstration that outcome of interest was not present at start of study | Study controls for  risk score | Study controls for any additional factor | Assessment of outcome | Was follow-up long enough for outcomes to occur (≥5 years) | Adequacy of follow up of cohorts |
| --- | --- | --- | --- | --- | --- | --- | --- | --- | --- |
| Lucas et al. 2009 | ★ | ★ | ★ | ★ | ★ | ★ | ★ | **-** | ★ |
| Hanfstein et al. 2014 | ★ | ★ | ★ | ★ | - | ★ | ★ | ★ | ★ |
| Jain et al. 2016 | **‐** | ★ | ★ | ★ | ★ | ★ | ★ | ★ | **-** |
| Lin et al. 2016 | **-** | ★ | ★ | ★ | - | - | ★ | ★ | ★ |
| Castagnetti et al. 2017 | ★ | ★ | ★ | ★ | ★ | ★ | ★ | ★ | ★ |
| Claudiani et al. 2017 | **-** | **-** | ★ | ★ | **‐** | **‐** | ★ | ★ | ★ |
| Pagnano et al. 2017 | - | - | ★ | ★ | ★ | ★ | ★ | - | ★ |
| Pfirrmann et al. 2017 | - | ★ | ★ | ★ | ★ | ★ | ★ | ★ | ★ |
| Rostami et al. 2017 | - | ★ | ★ | ★ | - | ★ | ★ | ★ | ★ |
| D’Adda et al. 2019 | - | ★ | ★ | ★ | ★ | - | ★ | ★ | ★ |
| Greenfield et al. 2019 | ★ | ★ | ★ | ★ | ★ | - | ★ | ★ | ★ |
| Sazawal et al. 2019 | - | ★ | ★ | ★ | - | - | ★ | - | ★ |
| Genthon et al. 2020 | ★ | ★ | ★ | ★ | ★ | ★ | ★ | ★ | ★ |
| Mulas et al. 2020 | ★ | ★ | ★ | ★ | ★ | ★ | ★ | ★ | ★ |
| Marce et al. 2021 | ★ | ★ | ★ | ★ | ★ | ★ | ★ | ★ | ★ |
| Shanmuganathan et al. 2021 | - | ★ | ★ | ★ | - | - | ★ | ★ | ★ |

Abbreviations: CML: chronic myeloid leukemia; CP: chronic phrase; IS: international scale; ★: yes; -: no

**Figure S1**. Funnel plots assessing publication bias.


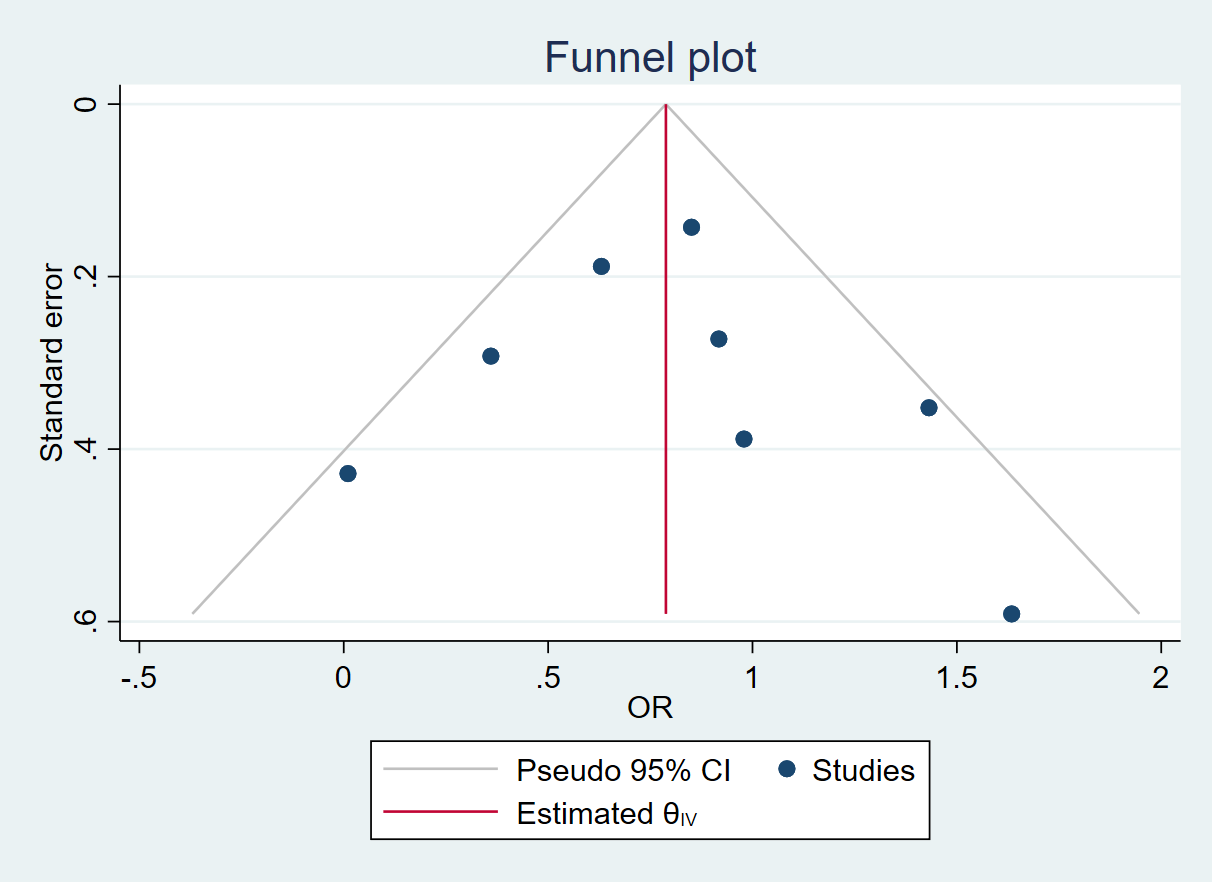


**Figure S2**. E14a2 versus e14a2 + e13a2: rate of patients who achieved major molecular response at 6, 12, 18 and 60 months.


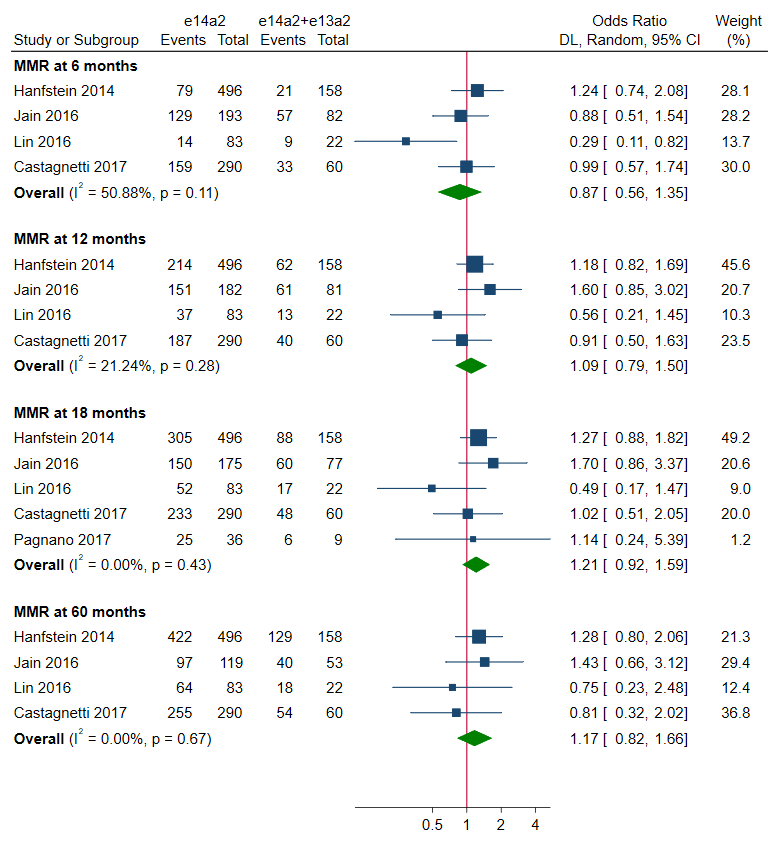


**Table S3.** Summary of meta-regression results for the 8 included studies

| Regression Variables | Number of Studies | Odds Ratio (95% CI) | P-value |
| --- | --- | --- | --- |
| Year of publication |  |  | 0.12 |
| ≤ 2019 | 5 | 2.47 (1.90-3.22) |  |
| > 2019 | 3 | 1.58 (0.95-2.61) |  |
| Sample size |  |  | 0.50 |
| ≤ 173 | 4 | 2.69 (1.35-5.33) |  |
| > 173 | 4 | 2.10 (1.72-2.55) |  |
| Criterion for DMR |  |  | 0.69 |
| MR4 | 5 | 2.32 (1.59-3.38) |  |
| MR4.5 | 3 | 2.07 (1.40-3.06) |  |
| Type of TKI therapy |  |  | 0.64 |
| imatinib | 4 | 2.08 (1.54-2.80) |  |
| second generation TKIs | 4 | 2.39 (1.43-4.02) |  |
| Sex (male/total) (%) |  |  | 0.55 |
| ≤ 53 | 4 | 2.44 (1.58-3.77) |  |
| > 53 | 4 | 2.05 (1.42-2.97) |  |
| Age (median) |  |  | 0.66 |
| ≤ 51 | 3 | 2.00 (1.15-3.46) |  |
| > 51 | 5 | 2.31 (1.66-3.22) |  |
| Median follow-up |  |  | 0.94 |
| ≤ 49 | 4 | 2.25 (1.41-3.60) |  |
| > 49 | 4 | 2.20 (1.50-3.22) |  |
| High risk (%) |  |  | 0.96 |
| ≤ 12 | 4 | 2.25 (1.49-3.38) |  |
| > 12 | 4 | 2.21 (1.46-3.35) |  |
| WBC (10^9^/L) |  |  | 0.23 |
| ≤ 71 | 3 | 1.85 (1.25-2.75) |  |
| > 71 | 3 | 2.47 (1.91-3.19) |  |
| Plt (10^9^/L) |  |  | 0.43 |
| ≤ 364 | 4 | 1.83 (1.20-2.78) |  |
| > 364 | 3 | 2.25 (1.67-3.04) |  |

Note: TKI, tyrosine kinase inhibitor; WBC, white blood cells; Plt, platelets; DMR, deep molecular response

**Figure S3**. E14a2 versus e14a2 + e13a2: rate of patients who achieved deep molecular response at 6, 12, 18 and 60 months.


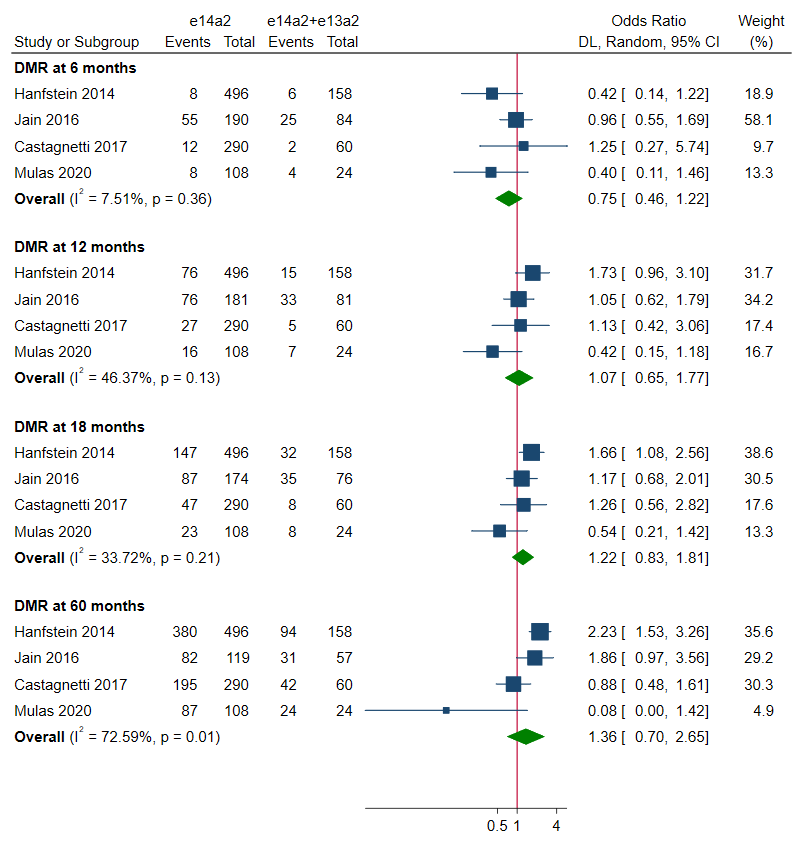


**Figure S4**. Survival outcomes for patients with the e14a2+e13a2 and e13a2 transcripts.


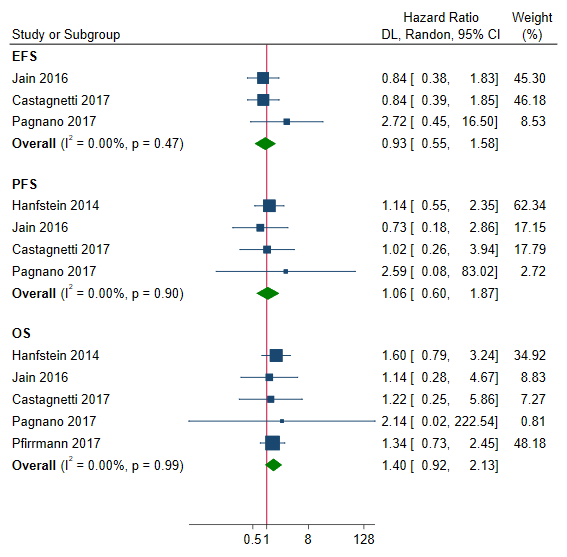


**Figure S5**. Survival outcomes for patients with the e14a2 and e14a2+e13a2 transcripts.


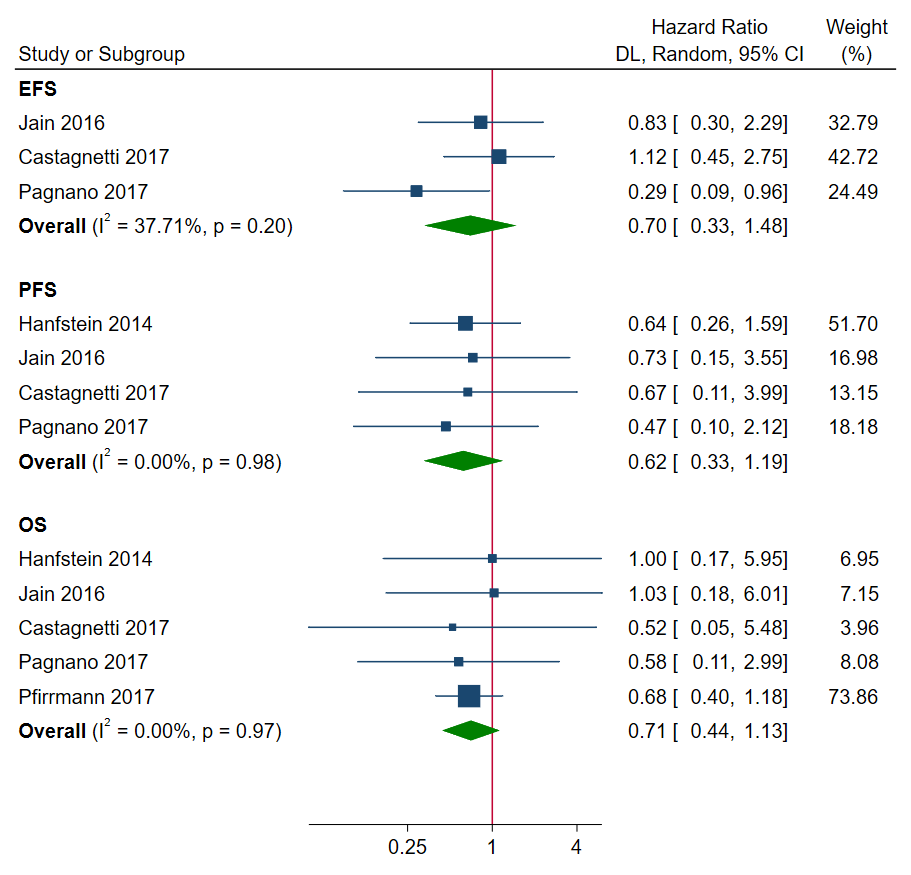


**Figure S6**. Network diagrams for the evaluated outcomes at 5 years. (A) major molecular response; (B) deep molecular response; (C) event-free survival; (D) progression-free survival; (E) overall survival. Each node represents a transcript type. The number of studies for each comparison is shown in the edges.


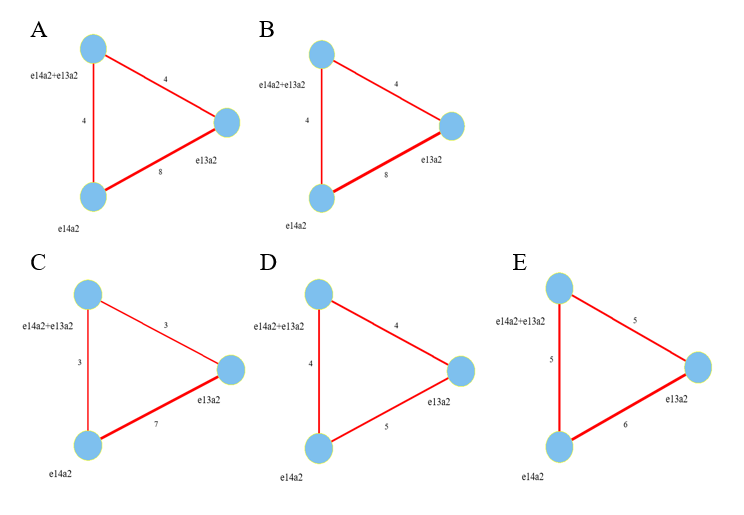


**Figure S7**. Odds ratios and 95% CrIs for major molecular response at 6, 12, 18 and 60 months between different transcript types. (A) 6 months; (B) 12 months; (C) 18 months; (D) 60 months.


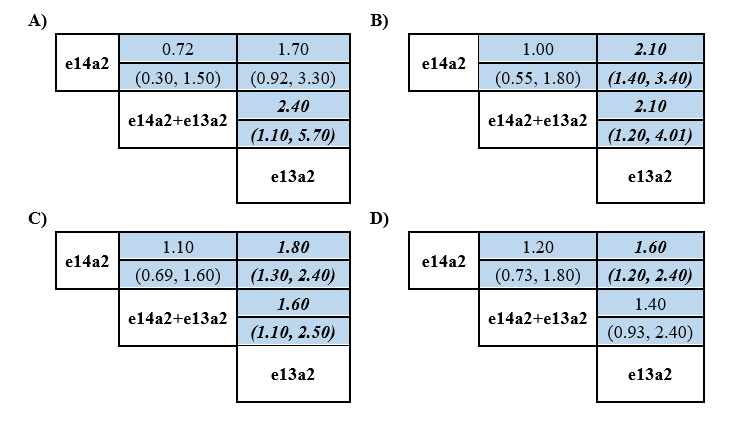


**Figure S8**. Odds ratios and 95% CrIs for deep molecular response at 6, 12, 18 and 60 months between different transcript types. (A) 6 months; (B) 12 months; (C) 18 months; (D) 60 months.


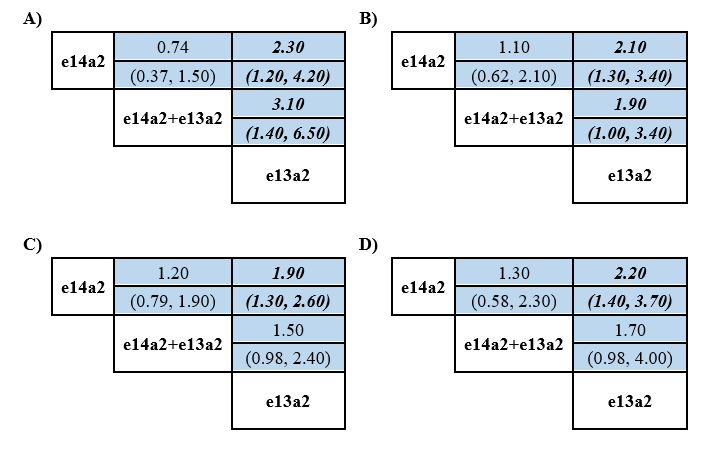


**Figure S9**. Hazard ratios and 95% CrIs for survival between different transcript types. (A) event-free survival; (B) progression-free survival; (C) overall survival.


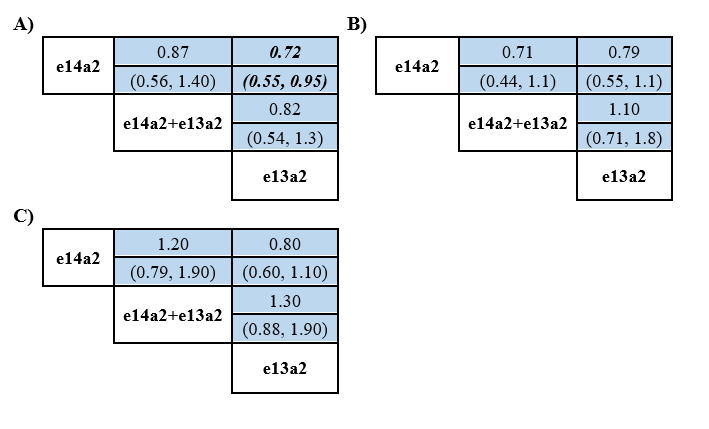

Supplement: Supplementary file 1 [file DataSheet_1.docx]
